# Rare Problems with Rotating Magnets in Cochlear Implants and How They Can Be Solved Without Surgery

**DOI:** 10.3390/jcm15093384

**Published:** 2026-04-28

**Authors:** Lutz Gärtner, Martin Zimmerling, Cornelia Batsoulis, Anke Lesinski-Schiedat

**Affiliations:** 1 Department of Otolaryngology, Hannover Medical School, 30625 Hannover, Germany; lesinski-schiedat.anke@mh-hannover.de; 2 Research & Development, MED-EL Medical Electronics, 6020 Innsbruck, Austria; martin.zimmerling@medel.com; 3 MED-EL Research Center, MED-EL Medical Electronics, 30625 Hannover, Germany; cornelia.batsoulis@medel.de

**Keywords:** magnetic resonance imaging, cochlear implant, rotating magnet, diametric magnetization, weakened magnet

## Abstract

**Objective**: To report on a series of three cases in which problems with rotating magnets (blocked rotation, demagnetization) occurred in cochlear implants and to resolve these problems without surgical intervention. **Methods**: Of the 3635 devices with rotating magnets implanted at this tertiary referral hospital, 2 exhibited rotation blockage (associated with misalignment of the coil or audio processor), and 1 was partially demagnetized in a 1.5 T MRI scanner. **Results**: One blockage resolved spontaneously without intervention. The second blockage was resolved in the static field of a 3T MRI scanner, where the demagnetized magnet was also re-magnetized to its original strength. Surgical intervention or re-implantation was not necessary in either case. **Conclusions**: Surgical intervention or re-implantation is not primarily required in the event of problems with the rotating implant magnet. Prior to surgery, technical analysis can lead to a conservative solution.

## 1. Introduction

More than one million people worldwide have a cochlear implant (CI) [1], a device that can restore speech comprehension in hard-of-hearing and deaf people through direct electrical stimulation of the auditory nerve. An externally worn audio processor with microphones and battery transmits signals to the implant via a coupling coil. Both the implant and the audio processor have a magnet so that the transmitter coil of the processor is aligned with the receiver coil of the implant and can thus be worn on the head.

Dependent on the implanted cochlear implant model, there may be limitations for CI users with regard to usable medical diagnostic methods. These include magnetic resonance imaging (MRI). Complications may arise from voltage induction in the implant, which could cause unintended stimulation and temperature increase. In addition, force and torque complications could occur due to the interaction between the implant magnet and the magnetic field of the MRI scanner [2,3,4,5]. In a 3 T MRI scanner in particular, there is an additional risk of demagnetization of the internal magnet [6,7]. Brain scans may be impeded due to imaging artifacts arising primarily from the implant magnet.

Nowadays, all modern CIs have a rotating internal magnet with diametric magnetization that aligns itself with the magnetic field of an MRI scanner, thus preventing demagnetization and significantly reducing the force exerted on the implant. In 2014, MED-EL (Innsbruck, Austria) was the first company to use a rotating magnet in its implants. Advanced Bionics (Valencia, CA, USA) followed suit in 2018 and Cochlear (Cochlear Ltd., Sydney, Australia) in 2019.

**Figure 1:**
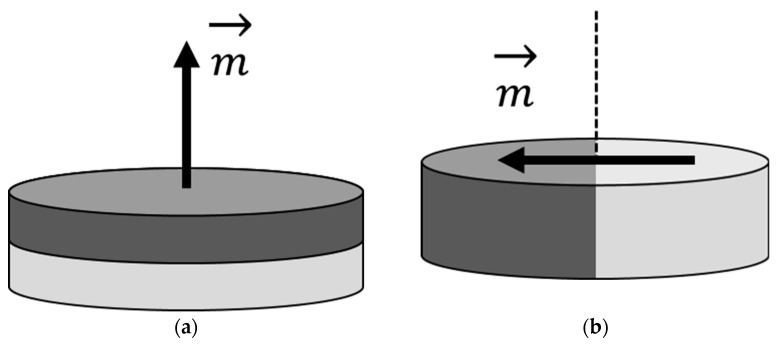
Magnetic disc (**a**) axially magnetized, (**b**) diametrically magnetized. The vector m shows the alignment of the magnetic dipole, which is directed from one pole to the other. The different shades represent the two different magnetic poles. A diametrically magnetized magnet is suitable for use as a rotatable magnet if it is mounted so that it can rotate along its axis (dashed line).

Figure 1 shows magnetic discs with axial and diametrical magnetization. With axial magnetization, the poles are located on opposite surfaces, while with diametrical magnetization, they are located along its diameter. In the magnetic field of an MRI scanner, an implant magnet can be both demagnetized and re-magnetized. This depends on the material used to manufacture the magnet but also on the strength and direction of the external magnetic field. In the case of non-rotating magnets, a large force is to be expected, which is why special precautions must be taken, such as fixation by means of a head bandage in accordance with the manufacturer’s instructions. Rotating magnets follow the external magnetic field if it is strong enough and if the dipole of the magnet is not coincidentally aligned antiparallel to the external magnetic field. Otherwise, weakening would also be possible.

## 2. Materials and Methods

### Subjects

At our tertiary referral hospital, we have implanted 3635 cochlear implants with rotatable internal magnets to date (as of the end of December 2025), and we report here on the rare problems encountered with them (Table 1).

## 3. Results

### 3.1. Problem 1: Rotation of the Internal Magnet Blocked

At the beginning of 2023, we noticed two cases in which the rotation of the magnet in the implant was blocked. These involved a Nucleus CI632 and a Nucleus CI622 from Cochlear. To the best of our knowledge, these are the first such cases to be reported.

#### 3.1.1. Case 1

A 67-year-old patient received a Nucleus CI622 on the left side. One day after surgery, early fitting took place with a KANSO2 (CP1150) sound processor. No irregularities were recorded. However, the patient did not wear the processor until the initial fitting phase, which took place 5 weeks later for 5 days. During the initial fitting, it was determined that the processor only stayed in place in a certain position, which was unfavorable for optimal use of the directional microphones. The patient considered re-implantation. Two months later, the patient came in for his next follow-up appointment. The processor could be worn as usual, and the internal magnet was freely rotatable again. The blockage had resolved without any apparent intervention.

#### 3.1.2. Case 2

A 70-year-old patient received a Nucleus CI632 on the left side. The initial fitting phase with an N8 (CP1110) sound processor took place six weeks after surgery. It was found that the transmitter coil could not be attached in any position—only one position was possible, with the cable end pointing upwards. The internal magnet in the implant could not be rotated. First, the external magnet was rotated approximately 180° out of the transmitter coil and then fixed with adhesive so that the processor could be worn as usual. At the next follow-up appointment 3 months later, the problem remained unchanged. It was discussed whether the lack of rotation of the internal magnet would now lead to a contraindication for an MRI. Finally, we decided on a controlled in-house examination, which was carried out another 4 months later, as the problem remained unchanged.

As a precaution, the patient was fitted with a head bandage and then pushed into the static 3 T field of one of our MRI scanners and immediately pushed back out again. The intervention therefore only took a few seconds. The internal magnet was then able to rotate freely again. The patient was able to maintain the original magnetic strength of the external magnet, i.e., no demagnetization took place.

### 3.2. Problem 2: Partial Demagnetization of a Rotating Magnet

#### Case 3

At the age of 29, a patient received a MED-EL CONCERTO with STANDARD electrode on the left side, which did not yet have a rotating magnet (Figure 1a). At the age of 38, he received sequential bilateral treatment on the right side with MED-EL SYNCHRONY2 FLEXSOFT. The latter CI already had a rotating magnet (Figure 1b). The patient uses a RONDO3 audio processor on both sides.

The patient needed an MRI of the abdomen, for which he was pushed feet first into a 1.5 T MRI scanner. With the older CI with non-rotating magnet, MRI can be safely performed up to magnetic field strengths of 1.5 T. Immediately after the MRI, there were no changes in the holding force on the left side. However, the processor on the right side no longer held. Increasing the external magnet from strength 3 to 5 resulted in a slight improvement. However, the retention force was still too low and the patient was very dissatisfied with this. Therefore, re-implantation was considered.

**Figure 2:**
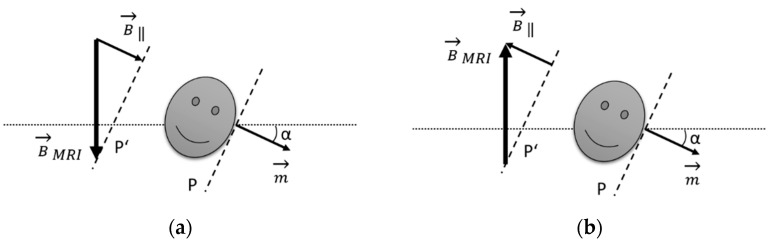
Position of the head during re-magnetization. To prevent the axial implant magnet from being weakened in the 3 T MRI scanner, the head must be tilted at an angle α (approx. 20–30°) to the shoulder. The direction of the external magnetic field of the MRI scanner B_MRI_ determines the side to which the head must be tilted. P, P′ = planes parallel to the pole faces of the axial implant magnet, whose magnetic dipole m is perpendicular to it. (**a**) Correct position. The portion of the external magnetic field B_‖_ = B_MRI_ sin(α) that is parallel to m has an amplifying effect. (**b**) Incorrect position. Here, B_‖_ and m would be oppositely directed—the magnet would be weakened.

The literature contains a report on the re-magnetization of an axial magnet that had been weakened by an examination with a 3 T MRI scanner and was successfully re-magnetized [8]. In our case, however, the rotatable diametrically magnetized magnet was affected. No such case has been reported to date. If only one implant with rotatable diametrically magnetized magnet were present, a straight head posture would be best for the re-magnetization. However, the presence of an axially magnetized magnet on the opposite side poses an additional challenge: not weakening that magnet. This can be achieved by tilting the patient’s head to the side by about 20 to 30° (ear to shoulder) so that the magnetic dipole moment of the magnet in the implant with a fixed, axially magnetized magnet has a component parallel to the static magnetic field of the MRI scanner [8]. See Figure 2 for details.

After consulting with the manufacturer, all parties (patient, implanting clinic, manufacturer) decided on a re-magnetization procedure, which had been successfully tested beforehand on a model using a clinical 3 T scanner. Even when the rotatable, diametrically magnetized magnet was oriented with its flat surface at an angle of 20–30° relative to the 3 T field, the magnetic dipole orientation remained unchanged after re-magnetization. It was also demonstrated that bringing an axially magnetized magnet into the magnetic field at this angle, where the magnet’s dipole has a parallel component to the 3 T field and not an anti-parallel component, does not weaken the magnet nor alter the angle of the magnetic dipole. This is possible because both implant magnets are anisotropic, meaning they can only be magnetized along a defined axis. Since no metallic objects, including sound processors, may be worn in the vicinity of an MRI scanner, the patient had to be given precise instructions, as communication could then only be nonverbal. The magnetic field alignment of the MRI scanner was determined using a transmission coil as described by Ropero Romero et al. [8]. This specified the direction in which the patient had to tilt his head before being moved into the scanner. In our case, the flat side of the coil that would be in contact with the skin on the head was facing towards the MRI scanner. Therefore, the head had to be tilted so that the ear with the CONCERTO implant was bent towards the shoulder. If the flat side of the coil had been facing away from the scanner, the head would have had to be bent with the CONCERTO side facing away from the shoulder.

A tight head bandage was applied to stabilize the left CI. The patient laid down on his back on the bed in front of the MRI scanner, with his head tilted to the left. The bed was then moved up in front of the tube and the patient was pushed head first into the static 3 T field of the tomograph and then immediately moved back again. The patient left the MRI scanner, and the retention force of his processors was checked. The RONDO3 for the left side was just as secure as before (magnet strength 3). The RONDO3 with magnet strength 5 held extremely well on the right and could be reset to its original strength 3. The actual procedure was successfully completed in a few seconds. Changes in speech comprehension were not expected, but were checked nonetheless. No impairments were found.

## 4. Discussion

Even before the introduction of rotatable implant magnets, it was possible to perform an MRI scan on some CI’s, provided certain precautions were taken, such as wearing a firm head bandage. However, there was still a risk of magnet dislocation, which usually can only be resolved surgically and meant that the patient was unable to wear the audio processor for a certain period of time [9]. In cases of pain during or after an MRI scan, Hassepass et al. [10] recommended an X-ray examination according to Stenvers for the early identification of magnetic dislocation after an MRI scan and called for caution when indicating an MRI scan for CI patients. The FDA published guidance on the feasibility of MRIs in CI without distinguishing between axially and diametrically magnetized implant magnets [11]. It is therefore understandable that some radiologists generally consider MRI to be contraindicated for CI patients—the risk seems too great. At our clinic, we are not aware of any cases involving dislocation of a rotating CI magnet, and Rupp et al. [12] have also not observed any such cases and therefore consider implants with diametrically magnetized magnets to be potentially safe with MRI under defined conditions.

Accordingly, it may happen that a demagnetized magnet is re-implanted, as this is a standardized procedure with known risks, while re-magnetization in an MRI scan appears to pose an unpredictable risk. With the cases presented here, we would like to show that a well-prepared re-magnetization procedure could be successful in a matter of seconds.

We were unable to determine the cause of the blocked rotation in the first two cases. In the first case, the problem was resolved spontaneously without further intervention approximately 3.5 months post-op. It can therefore be assumed that the blockage was not very severe. In case 2, the blockage was still present 8.4 months after surgery. In the static magnetic field of a 3 T MRI scanner, rotation was restored within seconds. Both patients had their last follow-up appointment in spring 2025, approximately two years after their surgery. In both cases, the implant magnet was able to rotate.

There are currently no objective measuring devices available for routine clinical use to measure the holding force of CI magnets. The assessment of the holding force is based on the experience of the clinicians and the history of the magnet force used by the patient before and after the MRI procedure. Since no swelling could be detected above the implant in case 3 and the audio processor was barely attached to the patient’s head, the only possible conclusion was that the internal rotatable magnet unexpectedly became partially demagnetized. Normally, a diametrically magnetized implant magnet rotates within its hermetic housing when exposed to a sufficiently strong external magnetic field, aligning parallel to this field. In this state, the implant cannot weaken; in fact, a strong external field can even strengthen a partially magnetized magnet. There is, however, a small chance the implant magnet will not align—specifically if its magnetic dipole is exactly opposite (180°) to the MRI scanner’s main magnetic field. In practice, the angle is almost never exactly 180° and varies during positioning in the scanner when the implant passes through fringing fields. Head movements can also slightly change this angle. Thus, the magnet usually rotates and aligns with the scanner’s static field. A very small probability remains that the magnet experiences no torque despite misalignment, namely when it is exactly antiparallel to the MRI field. We speculate that this was the reason for the magnet weakening in this case in the 1.5 T scanner. The field was strong enough to reduce magnetization but not to reverse it. In a 3 T scanner, any weakening would have gone unnoticed because the magnet would have immediately re-magnetized in the opposite direction without practical consequences.

Since the magnetic strength and the magnet’s ability to rotate cannot currently be objectively measured, it is not possible to verify that the original functionality has been restored as intended. Nevertheless, neither the CI recipients nor the clinical staff were able to detect any difference from the original condition.

Caution should be exercised with the re-magnetization methods described here, as this is a non-standardized procedure. We strongly recommend to consult the manufacturer before performing this procedure, particularly if the user has a hearing implant of a different brand in the other ear. All external parts of the implant system must be removed before the procedure. Finally, the procedure may only be performed if the skin over the implant(s) is intact.

## 5. Conclusions

Based on the number of CIs with rotatable magnets used at our clinic to date (Table 1), the occurrence of the described challenges—blockage of the rotatable magnet and partial demagnetization of the rotatable magnet—corresponds to a frequency of approximately 0.1%. However, due to the recent introduction of rotatable magnets and the growing number of CI patients who will require an MRI in their lifetime, this issue could become more prevalent and clinically significant. Diametrically magnetized CI magnets that have weakened magnetic strength or are blocked in their rotatability can be restored to their functionality in the static magnetic field of an MRI scanner. No imaging, radio frequency pulses, or switching of gradient fields are required. The procedure takes only a few seconds. However, it is not standardized and may therefore not be generally applicable. Nevertheless, our examples show that carefully considered non-standard procedures—verified with dummy implants, if necessary—can be successful in rare cases of problems with CI magnets. In this way, the patients in the two cases described were spared re-implantation—with its unpredictable outcome in terms of speech comprehension and the well-known risks of surgery.

## Figures and Tables

**Table 1:** Overview of CIs with rotatable internal magnets used at our clinic.

	MED-EL (Innsbruck, Austria)	Advanced Bionics (Valencia, CA, USA)	Cochlear (Sydney, Australia)
First used	July 2014	November 2018	May 2019
Implant types used	SYNCHRONY, SYNCHRONY2	HiRes ULTRA 3D	Nucleus CI600 Series, Nucleus CI1000 Series
Number	1654	530	1451
Magnet issues	1	0	2

## Data Availability

The original contributions presented in this study are included in the article. Further inquiries can be directed to the corresponding author.

## Abbreviations

The following abbreviations are used in this manuscript:

CI	Cochlear implant
MRI	Magnetic resonance imaging
